# A remotely piloted aircraft system in major incident management: concept and pilot, feasibility study

**DOI:** 10.1186/s12873-015-0036-3

**Published:** 2015-06-10

**Authors:** Håkon B. Abrahamsen

**Affiliations:** Department of Research and Development, The Norwegian Air Ambulance Foundation, 1441 Drøbak, Norway; Department of Anaesthesiology and Intensive Care, Stavanger University Hospital, 4011 Stavanger, Norway

**Keywords:** Remotely piloted aircraft system, Unmanned aerial vehicle, Situation assessment, Major incident, Pre-hospital, Avalanche, Human factors, Telemedicine

## Abstract

**Background:**

Major incidents are complex, dynamic and bewildering task environments characterised by simultaneous, rapidly changing events, uncertainty and ill-structured problems. Efficient management, communication, decision-making and allocation of scarce medical resources at the chaotic scene of a major incident is challenging and often relies on sparse information and data. Communication and information sharing is primarily voice-to-voice through phone or radio on specified radio frequencies. Visual cues are abundant and difficult to communicate between teams and team members that are not co-located.

The aim was to assess the concept and feasibility of using a remotely piloted aircraft (RPA) system to support remote sensing in simulated major incident exercises.

**Methods:**

We carried out an experimental, pilot feasibility study. A custom-made, remotely controlled, multirotor unmanned aerial vehicle with vertical take-off and landing was equipped with digital colour- and thermal imaging cameras, a laser beam, a mechanical gripper arm and an avalanche transceiver. We collected data in five simulated exercises: 1) mass casualty traffic accident, 2) mountain rescue, 3) avalanche with buried victims, 4) fisherman through thin ice and 5) search for casualties in the dark.

**Results:**

The unmanned aerial vehicle was remotely controlled, with high precision, in close proximity to air space obstacles at very low levels without compromising work on the ground. Payload capacity and tolerance to wind and turbulence were limited. Aerial video, shot from different altitudes, and remote aerial avalanche beacon search were streamed wirelessly in real time to a monitor at a ground base. Electromagnetic interference disturbed signal reception in the ground monitor.

**Conclusion:**

A small remotely piloted aircraft can be used as an effective tool carrier, although limited by its payload capacity, wind speed and flight endurance. Remote sensing using already existing remotely piloted aircraft technology in pre-hospital environments is feasible and can be used to support situation assessment and information exchange at a major incident scene.

Regulations are needed to ensure the safe use of unmanned aerial vehicles in major incidents. Ethical issues are abundant.

**Electronic supplementary material:**

The online version of this article (doi:10.1186/s12873-015-0036-3) contains supplementary material, which is available to authorized users.

## Background

**Fig. 1:**
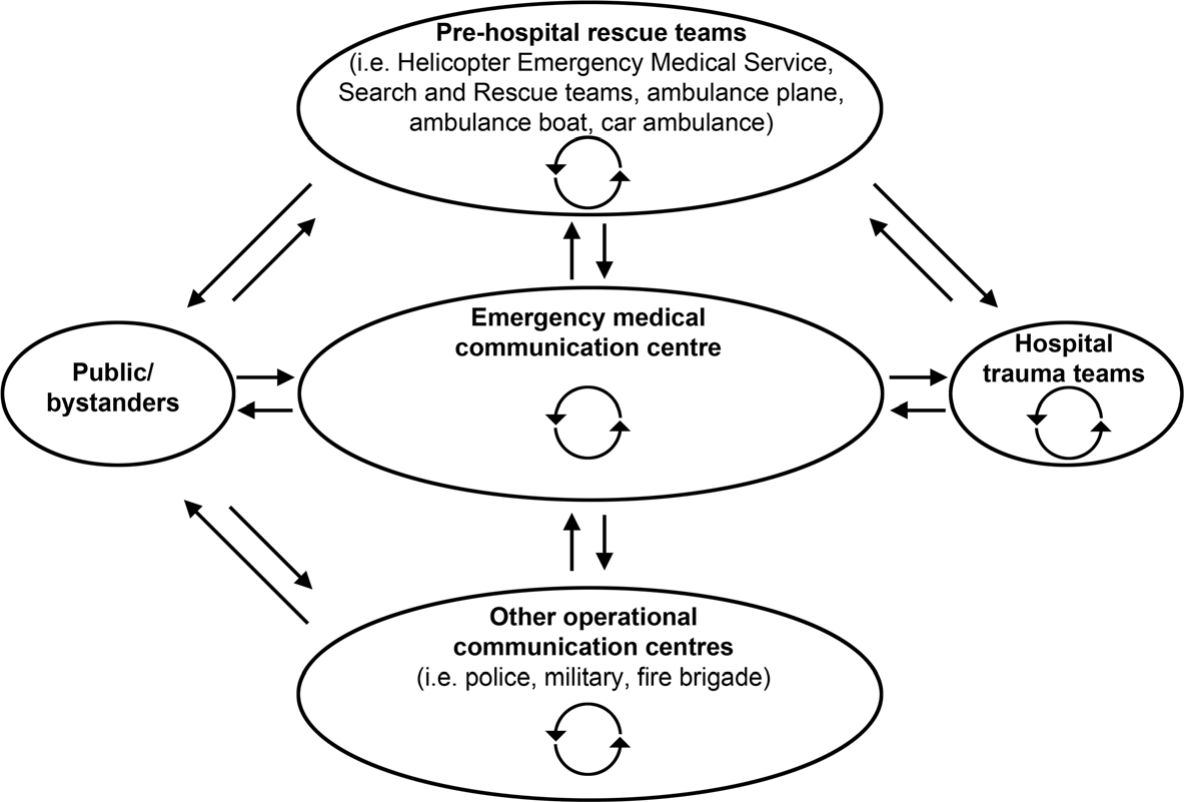
Typical communication links in major incident response in Norway. In the chain of pre-hospital emergency medical care, teams and team members are not usually co-located. In Norway, medical emergency calls from the public are handled by dispatchers at the local emergency medical communication centre who decide on the type of response needed [1]. The Joint Rescue Coordination Centres have overall operational responsibility during search and rescue operations. Together with the local emergency medical communication centres they coordinate and facilitate multidisciplinary cooperation and communication. Communication links, within and between teams, are depicted by curved and straight-lined bidirectional arrows, respectively. (Illustration: Kim Søderstrøm)

In Norway, communication and information sharing in pre-hospital environments is primarily voice-to-voice through phone or radio on specified radio frequencies. There is no visual aid to help rescue teams and the local emergency medical communication centre to understand complex scenarios (Fig. 1) [1]. Barriers to communication in pre-hospital environments are diverse by nature [2]. Errors can be made both in speaking and hearing. Critical information is often missing [3–5].

Shortly after a terrorist attack, a natural disaster or a mass casualty event, the disaster area is chaotic, complex and unclear; infrastructure may be destroyed and the scene often spans large distances [6–8]. The aim of the immediate pre-hospital medical response in major incidents like this is to localise, triage, treat and evacuate casualties in an organised and efficient manner [6]. In order to take appropriate action, decision-makers on the scene and at the emergency medical communication centre need to build a mental model of what has happened, what is going on and what the problem is [1]. In operational settings we use the term ‘situation assessment’ [9, 10].

Situation assessment is critical for decision-making and for safe and efficient task performance [5, 11, 12]. In major incidents, decisions have to be made under conditions of inadequate or ambiguous information, high uncertainty and intense time pressure [13]. The task environment is characterised by ill-structured problems, shifting goals and high costs for failure. Healthcare providers can experience this as obstacles to gathering information and integrating findings [14, 15]. It can be difficult for healthcare providers to obtain an overview of what is going on; this is due to rescue workers’ limited field of view from the ground, and the myriad of parallel events in an unfolding situation, spread over large, geographic areas in challenging terrain. Physical distance between the incident commander and rescue teams or rescue team-members on the scene is a barrier to direct communication and to many other aspects of teamwork [16]. First responders can face hazards such as ongoing shooting, radiation, infectious and chemical agents, explosion hazard, fire, smoke or gases.

Aerial imagery and remote sensing in general have been used by the military for many years to provide support to people on the ground in decision-making, situation assessment, reconnaissance and surveillance [17, 18]. Satellites, airships and manned aerial remote sensing have important limitations [19]. High resolution satellite imagery from most satellite providers is restricted by mist and cloud cover. Manned aerial remote sensing undertaken by a crew on board a helicopter or airplane is costly, and human safety can be compromised.

Rapidly maturing technologies like unmanned aviation, wireless technology and the miniaturization of high-resolution imaging systems have resulted in the proliferation of civilian and recreational applications of remote sensing [18, 20, 21]. Few studies have been conducted on pre-hospital and medical applications of remote sensing from low altitude in major incidents. Aspects of wilderness and urban search and rescue using unmanned aerial technology have been studied [22–24], but most of the papers have a technical content and focus of interest that makes them unavailable to anyone other than a technical audience [25]. Remotely piloted aircraft (RPA), sometimes referred to as unmanned aerial vehicles (UAV) or unmanned aerial vehicle systems (UAS), are commercially available off-the-shelf in a variety of different sizes and configurations [21]. They can be equipped with sensors, although they have a limited payload capability. They can be custom-made to the desired specifications and performance requirements and can be classified as either rotor-wing (helicopter) or fixed wing (airplane).

The aims of this study were 1) to assess the technical feasibility of using an unmanned, remotely piloted aircraft as a tool carrier for audiovisual equipment and sensors in pre-hospital environments and 2) to test the feasibility and concept of remote situation assessment in simulated major incidents by using unmanned aerial vehicle remote sensing to gather and distribute information.

## Methods

### Conceptual framework

**Fig. 2:**
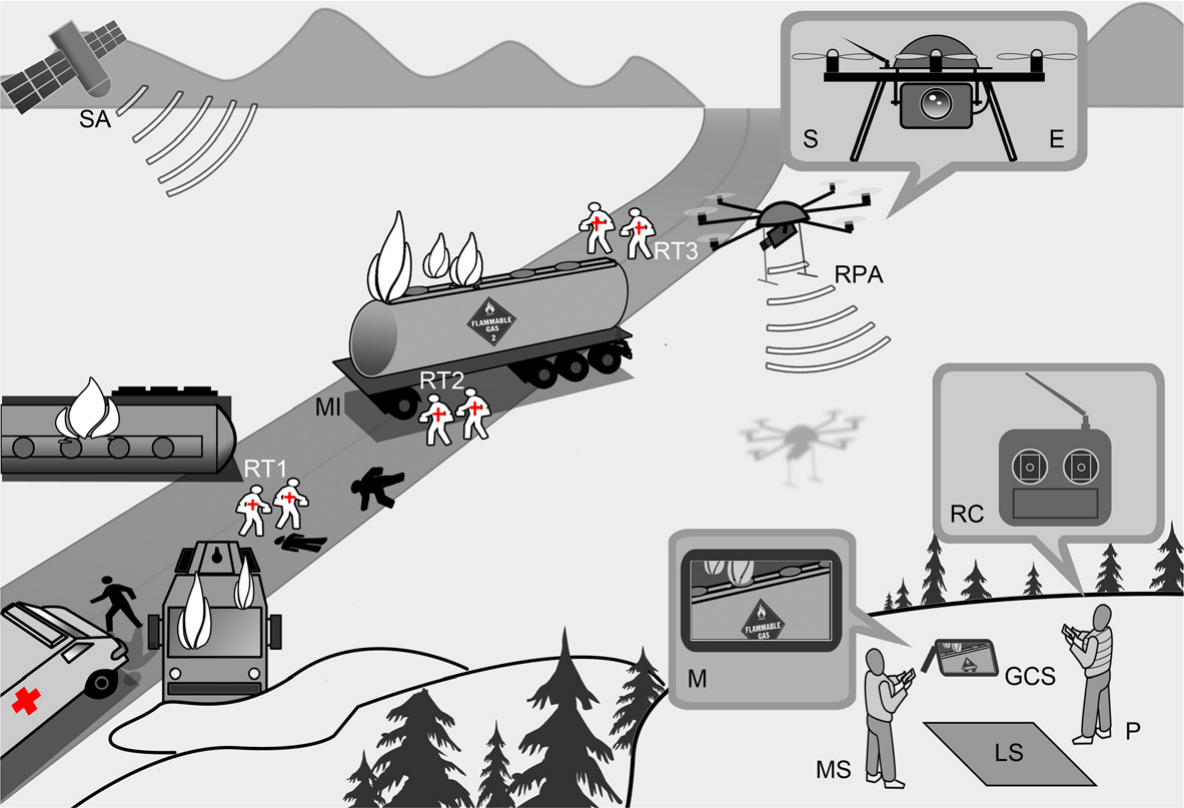
Concept of line-of-sight operation of a remotely piloted aircraft system in major incident management. A bus and a truck with flammable gas on fire after a collision. The unmanned remote piloted aircraft (RPA) is under the control of a remote pilot-in-command (P) on the ground assisted by a mission specialist (MS). Signals from satellites (SA) together with on-board automation such as a GPS module and a micro controller unit help to manoeuvre the vehicle. Real-time aerial video of the major incident (MI) scene is streamed to a ground control station (GSC) and displayed on the monitor (M). Pre-hospital rescue teams (RT1-3) that are not co-located treat injured trauma patients (black). (Illustration: Kim Søderstrøm)

A concept sketch of the remotely piloted aircraft system is shown in Fig. 2. An RPA was used as a flying platform for sensors (S) (video camera, avalanche beacon) and as a tool carrier for equipment (E) (laser, release hook, searchlight). A small helicopter was considered suitable for the task because it can perform vertical take-off and landing, hover, make sharp turns and manoeuvre with high precision at low speed in confined spaces. Any flat, solid ground measuring at least 1x1 m was suitable as a landing site (LS). Flight control was mixed manual (remote control (RC)) and autonomous (autopilot). The RPA pilot (P) was experienced and approved by the Civil Aviation Authority (CAA). He remotely piloted the aerial vehicle into a position above the scene of a simulated major incident (MI) within line-of-sight. Autonomous flight was controlled by a microcontroller unit and a Global Positioning Module (GPS) on board the aircraft. Live aerial video and data from the sensors was wirelessly transmitted in real time to a ground control station (GCS) and displayed on a monitor (M). Data regarding the positioning, speed and altitude of the RPA overlaid this video. A mission specialist (MS) with three years of pre-hospital experience with air ambulance services in Norway and seven years as an anaesthesiologist assisted the pilot to interpret the output on the screen, frame the video shots and direct the RPA into position.

### The RPA platform

**Fig. 3:**
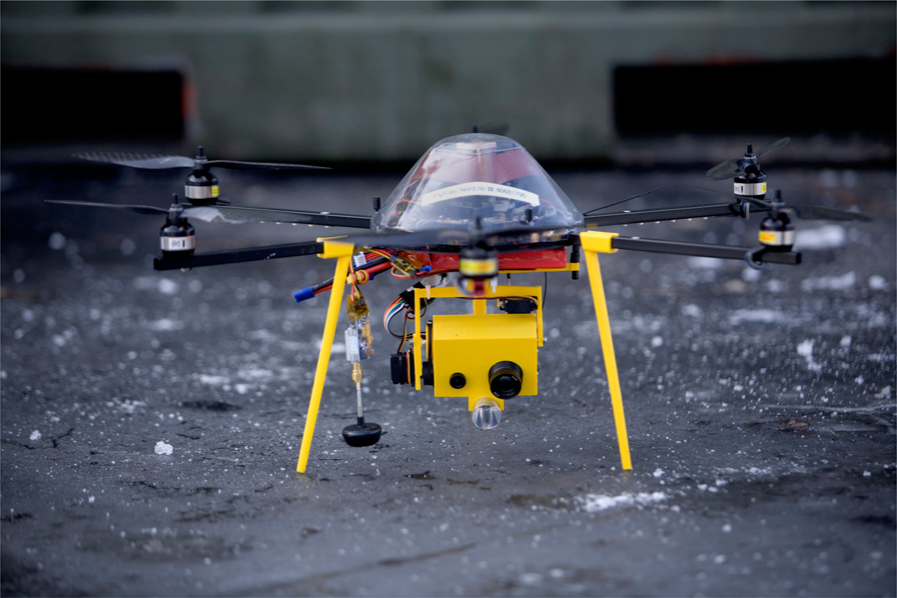
Remotely Piloted Aircraft (RPA). Radio controlled multirotor unmanned aerial vehicle with searchlight and video camera. (Photo: Adrian Johansen)

An experimental multirotor RPA with vertical take-off and landing was customized for use in pre-hospital environments with autonomous features like steady hover and automatic landing (Fig. 3). The RPA was propelled by six standard brushless electric (DC) rotors. The rotorspan was 84 cm and maximum take-off weight was 3 kg. Vehicle motion was controlled by altering the rotation rate of one or more of the rotor discs. The DC motors were powered by a rechargeable lithium-ion battery pack, giving an approximate runtime of 15 min. A power supply (220 volt) was available from a generator in a fire truck, and batteries could then be charged and swapped using a portable battery charge unit and an AC/DC converter. The support frame and landing gear were 3D-printed out of a thermoplastic aliphatic polyester called Poly Lactic Acid (PLA). The RPA was remotely controlled by using a 2.4 GHz frequency hopping spread spectrum remote control. A wireless 5.8 GHz transmitter was used for video downlink to the ground station. The electronic circuits were covered by an orange plastic cap for greater visibility in the snow.

### Sensors and equipment

Maximum payload capability was 1.54 kg. A pivotal support, which allowed rotation about the horizontal axis, enabled easy mounting of the following: a small high-definition (1080p at 60 frames per second) action camera (GoPro Hero 3, Black Edition) with a 170-degree field of view and a 1050-mAh lithium-ion battery; a digital daylight colour camera (768x492 pixels); an infrared camera (640x512 pixels) with built-in power supply from the RPA; a laser beam and a searchlight. In order to avoid electromagnetic interference, a 50-cm hinged suspension system for a three-antenna 457-kHz avalanche transceiver (Mammut PULSE Barryvox) was used to keep it away from electronics and magnetic objects in the RPA. The pilot remotely controlled camera tilt and a mechanical gripper arm which could be opened and closed.

### Setting and environmental conditions

We carried out a series of full-scale (Scenarios 1 and 2) and half-scale (Scenarios 3–5) outdoor pre-hospital exercises in March 2013. These took place on 13 March in Moss (Scenarios 1 and 4) and on 25 March in the mountains of Setesdalen, Hovden, in Norway (Scenarios 2, 3 and 5). It was intended that each of the exercises described below should reflect operative issues in pre-hospital critical care and disaster management.

The RPA was airborne and controlled within line-of-sight in all lighting conditions ranging from dawn to bright daylight to night. Air temperature ranged from +8 °C at noon to −7 °C in the evening. Wind speed varied from calm (Scenarios 1–5) to gentle breeze with stronger gusts of wind and turbulence (Scenario 3). Visibility was excellent; the sky was clear without precipitation in all scenarios. In the mass casualty traffic incident scenario, a powerful transmitting radio antenna was located on the rooftop of a fire station 100 m away from the exercise. Obstructions to air navigation were high voltage cables and trees in the avalanche scenario, buildings and vehicles in the mass casualty traffic accident scenario and steep mountain walls in the mountain rescue scenario. The airspace in all five scenarios was uncontrolled. This means that no clearance from air traffic control was required. The RPA was grounded in the case of nearby helicopter traffic.

### Simulation Exercise 1: A mass casualty traffic accident

A multiple-vehicle collision was set up as a full-scale simulation outdoors on an open parking lot. This was part of an interdisciplinary emergency service cooperation course (TAS), in which healthcare, police, fire and rescue technicians are taught self-safety, triage, patient evacuation, extrication techniques and cooperation through major incident simulation and practical sessions [26]. Responders from multiple rescue services in Norway and Sweden attended. Two cars were placed in close proximity to an articulated bus which was overturned. Real-sized car wrecks were used. Twenty-five schoolchildren were placed inside the bus to simulate injured and entrapped passengers. During the exercise the emergency response personnel were instructed to apply the principles of major incident management they had learned on the course. The RPA was controlled from a nearby position on the ground behind the bus, outside the rescue zone, in order not to interfere with the rescue exercise. A daylight- and an infrared camera were used.

### Simulation Exercise 2: Mountain rescue

**Fig. 4:**
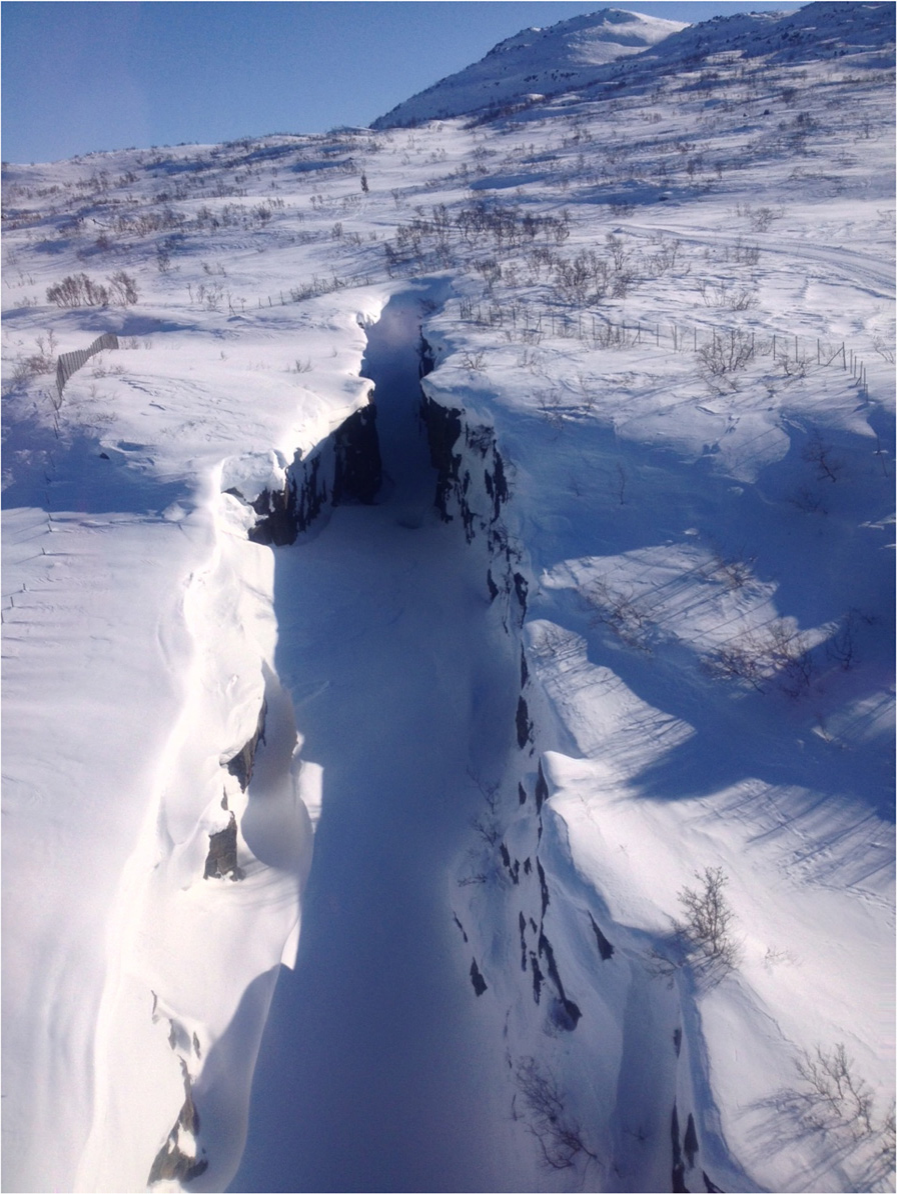
Airview of narrow canyon. A narrow canyon with steep rock faces and snowdrifts hanging over the edge was chosen as the location for a mountain rescue scenario. Note the big hole in the snow at the bottom of the canyon with running water beneath, not visible from the edge. Photo shot from inside a manned helicopter (EC 135). (Photo: Håkon B. Abrahamsen)

A narrow canyon with steep rock faces and snowdrifts hanging over the edge was chosen as the location for a mountain rescue scenario (Fig. 4). The canyon was situated next to a groomed ski trail. The river bed at the bottom of the canyon was covered in snow and thin ice with running water beneath. The weight-bearing capacity of the snow and ice was tested and was found insufficient to support a human, making it unsafe to walk or drive across the riverbed with a snowmobile.

A manned ambulance helicopter (EC135 P2+) was flown over the canyon at various altitudes for reconnaissance and in order to prepare for an underslung rescue operation. This overflight was carried out with no individuals on the canyon riverbed for safety reasons. The researcher filmed the canyon from inside the helicopter with an action camera with a wide-angle lens. The helicopter crew provided feedback on how the exercise was to be carried out.

Afterwards, a simulated injured skier was positioned at the foot of the mountain wall and was instructed not to move his legs, as if injured and trapped. He was secured with ropes and wore a helmet. An alpine rescue team planned for and conducted the extrication of the patient with a sled. A Norwegian television team filmed the rescue operation with conventional cameras from the ground. The RPA was located on a ledge about 50 m away from the patient and was equipped with a high definition camera.

### Simulation Exercise 3: Unknown number of skiers buried in an avalanche

**Fig. 5:**
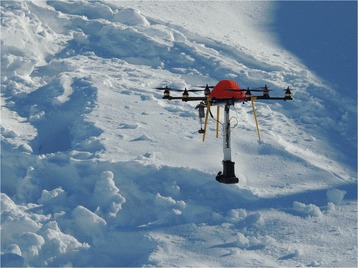
Avalanche beacon search from fixed altitude above the ground using a remotely piloted aircraft. A 50-cm hinged suspension system for a three-antenna 457-kHz avalanche transceiver (Mammut PULSE Barryvox) was used to avoid electromagnetic interference. (Photo: Håkon B. Abrahamsen)

A gently rolling area with deep, loose snow measuring approximately 50 × 50 m at the bottom of a ski resort was used to simulate a small avalanche. The area was cordoned off. Two avalanche beacons were borrowed from the local mountain rescue team. They were activated in “SEND” mode and placed in separate backpacks, which were buried in the snow approximately 50 cm below the surface and 20 m apart. Tracks in the snow were then wiped out in order to blind the location of the buried beacons to the RPA pilot and the mission specialist, who were located just beyond the outer boundaries of the avalanche. They were told that an unknown number of skiers were buried. The three-antenna avalanche transceiver (Mammut Pulse Barryvox) was put in “SEARCH” mode and attached beneath the RPA by a hinged arm to avoid interference from metal parts and electronics. The RPA was piloted within line-of-sight at a fixed altitude above the ground (Fig. 5). The mission specialist guided the pilot based on visual turn directions and aural cues from the ground station.

After the ski lift was closed, a similar exercise was carried out at the top of the ski resort in order to introduce more wind and turbulence into the scenario.

### Simulation Exercise 4: Ice fisherman through thin ice in a lake

A person was instructed to lie flat on the ice approximately 50 m from shore, simulating that he had broken through thin ice. The RPA pilot and the ground station were located on shore 50 m away, and the RPA was piloted within line-of-sight. A small bag of equipment, with similar weight to that of a couple of ice spikes, was attached to the mechanical claw before take-off.

### Simulation Exercise 5: Aerial search for casualties under different lighting conditions

The RPA was piloted within line-of-sight in daylight and using its navigation lights and the on-board infrared camera at night. A searchlight lit up the terrain below in the dark and a powerful green laser beam pointed out objects on the ground.

### Data collection

A combination of direct observation using audio- and video recordings, informal interviews, participant observation and researcher’s field notes was used in a mixed methods approach. After simulation exercise 1 was completed we asked actors and rescue personnel how they perceived the presence of the RPA in the air. Noise levels generated from the RPA were not measured on location in order not to interfere with the simulation exercises. In simulation exercise 2 we made a qualitative assessment of whether background noise from the RPA overlaid the voices of rescue personnel on the radio and in this way disturbed signal transmission. A photographer on the ground took still images of the simulated mass casualty traffic accident. Video from the daylight camera and from the thermal imaging camera was saved on a memory card at the ground station. High definition recordings from the action camera were saved on a micro Secure Digital (SD) memory card in the video camera itself, while a down-scaled recording was transmitted simultaneously to the ground station and displayed in real time. The researcher (HBA) engaged in field work in all simulation exercises. Informal interviewing of the observers was performed as part of the process of observing. One of the course participants in simulation exercise 1, a Swedish fire ground commander, observed parts of the simulation from the ground station. He gave feedback on how the RPA was used in this scenario and shared his ideas on how the RPA could be used in a real incident. An independent RPA expert from the Norwegian Board of Technology observed simulation exercises 1 and 4. A mountain rescuer observed simulation exercise 3 at the top of the ski resort. The RPA pilot shared his experiences continuously. Informal participant feedback was obtained and interviews with actors and rescue personnel were carried out after the simulation exercises were finished to gain insights and perspectives. Field notes were written after leaving the field site.

### Ethics

This study did not fall under the mandate of the Regional Committee for Medical Health Research Ethics in Norway [27]. Participation was voluntary and oral informed consent was obtained in advance. No real patients were included; only healthy individuals were used to simulate trauma victims in the scenarios. The rescue workers involved were all professionals working in rescue services in Norway and Sweden. Both actors and professionals were given the opportunity to withdraw from the study at any time and without stating any particular reason. Participants were informed that video and still images would be shot from both the ground and the air during the simulations and that video and still images were only to be used in accordance with the purpose of the study. None of the participants could be identified or recognised in the results of the study. Data could not be used to evaluate the skills of the course participants or as feedback to their management. The safety of the participants in the scenarios was assessed and ensured by competent rescue personnel not directly involved in the simulations. All participants were required to use proper protective clothing and equipment; naked skin was barely visible. The risk and inconvenience to the participants was considered minimal.

## Results

The RPA was partially dismantled before transportation to the location. It was small enough to fit into a medium-sized carrying case for protection. On location the RPA was assembled and prepared in less than five minutes. The diameter was 84 cm fully assembled. Depending on the payload, gross weight ranged from 1.7 kg to 1.9 kg. A battery pack kept the RPA airborne for about 15 min. Exchange of rechargeable battery packs was carried out in a few seconds and made it possible to keep the aircraft airborne almost continuously.

In all scenarios the RPA was remotely controlled within line-of-sight at different altitudes and speeds. The presence of the RPA flying over the simulated incidents in exercises 1 and 2 was not perceived as unpleasant or disturbing by the actors and rescue operators.

Noise from the RPA was limited. Rescue workers in simulation exercise 2 perceived the sound from the rotors as a slight buzz not louder at its maximum than the sound level of a normal conversation. Noise from the RPA did not overlay voices on the radio used for communication between rescue workers.

Real-time aerial video (resolution 720 × 480) from the scene was successfully transmitted wirelessly from the RPA to the ground station and displayed on a monitor. In calm air the RPA could be positioned with high precision. Wind gusts and turbulence greatly affected the operation of the RPA and made it challenging for the pilot to maintain a steady hover and fixed altitude above the ground. Turbulence and the RPA’s rapid altitude changes in windy conditions made jittery motions in the video recordings. Electromagnetic interference disturbed signal reception when the RPA was flown in the direction of a transmitting antenna. Short duration pulses of signal noise reduced the image quality but never impaired interpretation of the video streamed to the base.

Specific results from each of the exercises are presented below.

### Scenario 1: A mass casualty traffic accident

**Fig. 6:**
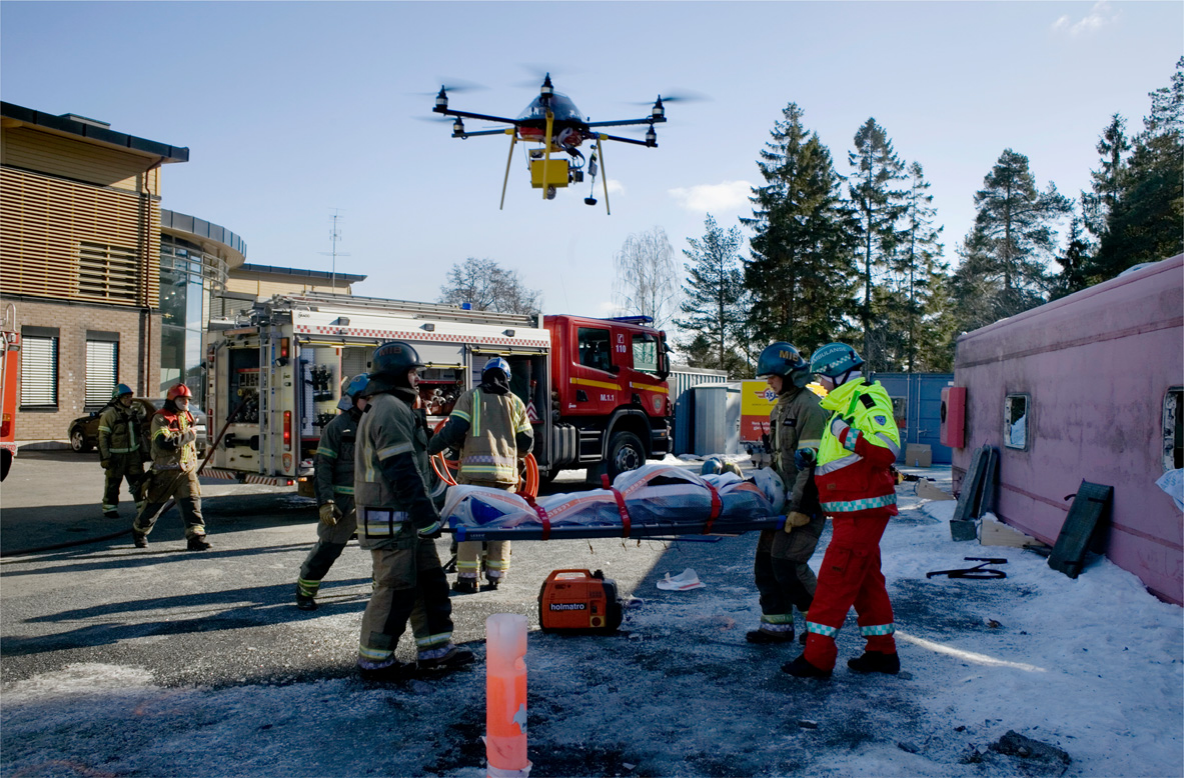
Remote sensing from a simulated major incident. Remotely piloted aircraft flying over a simulated major incident. A multiprofessional rescue team (front) working on the evacuation of a trauma patient from an overturned bus (right). The transmitting antenna that caused electromagnetic interference can be seen on top of the roof in the background. (Photo: Adrian Johansen)

The RPA was airborne before the first responders arrived at the scene. Based on live aerial video from 60 to 80 feet above the ground, it was possible to determine what kind of accident had happened, how many vehicles were involved and the extent of damage to the vehicles. From a low altitude (Fig. 6) and through the broken windshield in one of the cars involved, an unconscious trauma victim was identified sitting in the front seat without his seat-belt on. His head was tilted forward against the steering wheel and no respiration movements were detected. The RPA was not small enough to be flown into the bus or the car wrecks. Through an open hatch on top of the overturned bus, the infrared camera captured images of passengers lying and standing in the dark inside the bus. When rescue units arrived, it was possible to distinguish the different professions based on their uniforms and helmets. Allocation of resources, order of measures and treatment, number and flow of evacuated patients were monitored from the ground station.

### Scenario 2: Mountain rescue

**Fig. 7:**
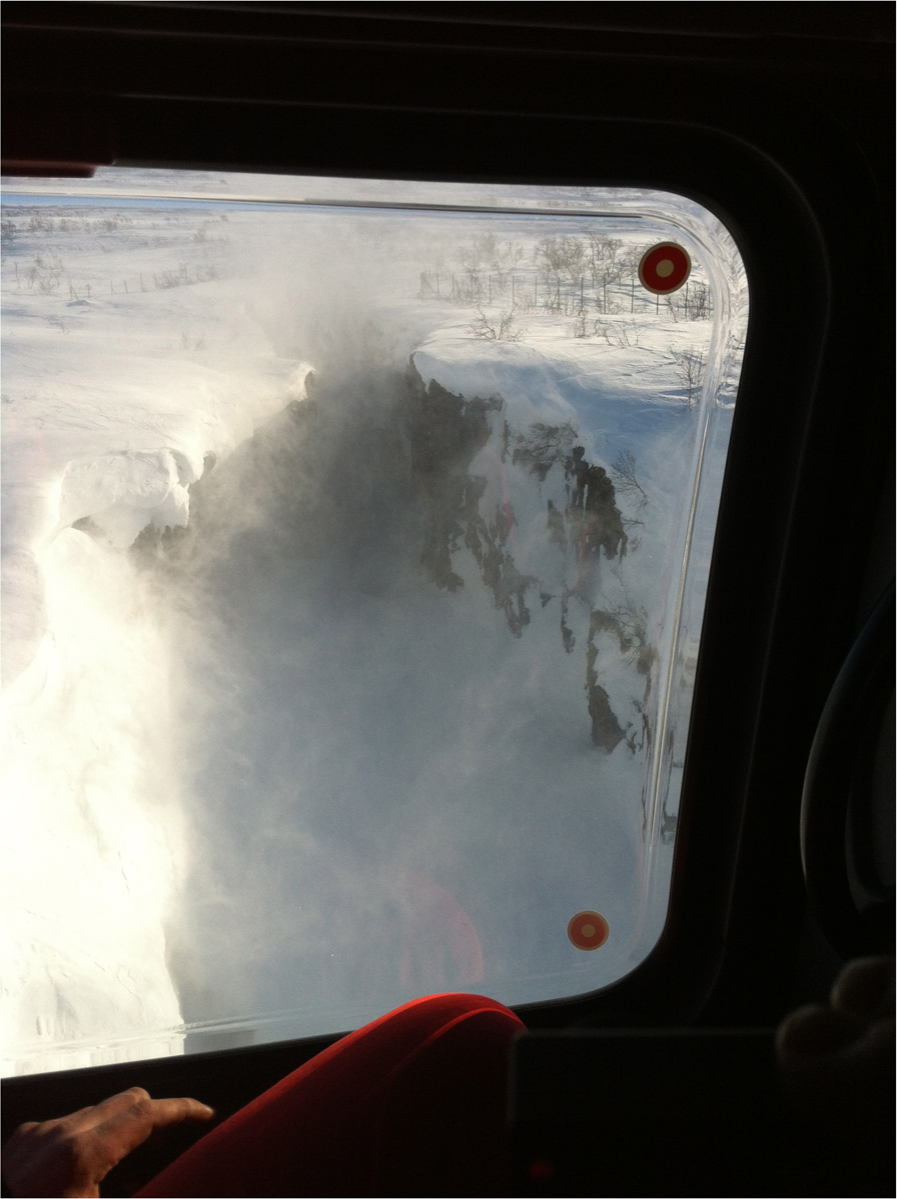
Low level flight over canyon. Low level flight over canyon with manned helicopter (EC 135). Downwash causes snow to swirl up, thereby restricting visibility. Photo shot from inside the helicopter. (Photo: Håkon B. Abrahamsen)

The level of detail in the aerial video shot from more than 100 feet from the RPA was similar to that in the video shot from a manned helicopter and provided no additional information. However, the video shot from the manned helicopter was free from jittery motions. At lower altitudes the rotor downwash from the EC135 started to cause snow to swirl up (Fig. 7). At 50 feet above the canyon, the downwash heavily kicked snow up into the air. This reduced visibility in the canyon significantly and made it difficult to spot details on the ground. The downwash also made large pieces of snow and ice break off from the snowdrifts on the edge. Downwash from the rotors on the RPA was negligible and could not be felt from a distance of more than six feet.

The RPA was flown into the canyon and positioned at different altitudes above the injured skier (Additional file 1). Respiratory movements and frosty breath from his mouth in the cold air were seen on the monitor in the ground base. The skier was alert and moved his head. No other injuries or large deformities were identified in his legs or arms. A big hole in the snow and ice on the riverbed and longitudinal cracks in the overhanging snowdrifts, not visible to the pilot from the edge of the mountain, were identified. It was possible to observe rescue climbers planning and conducting the evacuation of the skier.

### Scenario 3: Unknown number of skiers buried in an avalanche

A remote aerial visual search for survivors was performed from different altitudes. Use of a daylight video camera detected loose objects such as gloves and skis in the snow. An aerial visual search and aerial avalanche beacon search could not be performed at the same time because of technical limitations. The avalanche beacon search required a minimum of two operators. The pilot had to maintain a fixed altitude close to the ground, while systematically searching the surface of the avalanche until an analogue signal was picked up. From where the signal was first detected, the pilot was guided by the mission specialist in the right direction. Turn directions (arrows) and relative distance to the buried subject, indicated by numbers in the display, were wirelessly transmitted from the transceiver to the ground base and orally communicated to the pilot. Search sound and tone volume were also audible to the pilot, which accelerated the search. The pilot maintained a steady hover in close proximity to the buried subject and rescuers were sent to the location of the RPA in order to pinpoint the position and dig it up.

### Scenario 4: Ice fisherman through thin ice in a lake

The RPA was used as a tool carrier. A small cargo net of equipment was attached to the mechanical hook underneath the RPA, transported and dropped within reach of a person lying on the ice.

### Scenario 5: Search for casualties in complete darkness; aerial search for casualties under different lighting conditions

The RPA was lifted in an open area in complete darkness at night. The pilot did not use night vision goggles. A searchlight lit up the ground and a powerful green laser beam pointed out objects in the snow. An infrared camera detected human body-sized silhouettes and warm objects.

## Discussion

We have shown that a small remotely piloted aircraft can be used as an effective tool carrier, although limited by its payload capacity, wind speed and flight endurance. Remote sensing, using RPA technology already existing in pre-hospital environments, is feasible and can be used to support situation assessment and information exchange at a major incident scene.

### Operational benefits

An RPA with audiovisual equipment and sensors attached is a flexible, low-cost, time-efficient tool. It can safely and accurately support the understanding of what is going on in the field during major incidents involving infrastructure and telecommunication breakdown, unstable security settings for rescue workers and poor weather conditions.

More than 60 % of rescue missions following an avalanche pose considerable danger to ground searchers [28]. An RPA can make it possible to conduct a more targeted search. Visual cues from a video camera and turn directions from an airborne avalanche beacon can be distributed to decision-makers on the ground, such as incident command, rescue teams and the local emergency medical communication centre. Large distances can be covered in a short time with an RPA. This can be particularly useful when searching for victims of large avalanches, since victims visible on the surface have the highest probability of survival [28]. More than half of the victims are partially buried and visible on the surface. Survival decreases rapidly with time to extrication [29].

Downwash and noise from manned helicopters involved in search and rescue operations [30] and in medical response to major incidents may impair the operation itself. Noise on the ground from a manned helicopter (EC-135) hovering at approximately 100 feet during an underslung rescue operation exceeds 85dBA [31]. When rescue workers are exposed to noise levels of this magnitude, hearing protection is required to avoid noise-induced hearing loss. Communication by conventional radio or telephone without active noise reduction is almost impossible.

An avalanche beacon search from a low altitude using a manned helicopter can make it more difficult for ground searchers to perform simultaneous searches for buried victims using search and rescue dogs (Additional file 2) [32]. These dogs detect human scent. Powerful gusty downwash disperses the scent quickly in all directions. Both dogs and humans can be distracted by the loud noise, and communication between rescue workers will be impaired because they need hearing protection. Swirling and drifting snow will restrict visibility severely and make a visual search difficult from both the ground and the air. Small and loose objects in the snow may blow away or be buried in the swirling snow. Downwash and noise from an RPA is minimal and will to a much lesser extent disturb and inhibit rescue workers and dogs from doing their job.

In our mountain rescue scenario, downwash from the EC135 blew large blocks of snow down into the canyon. This could have represented a serious hazard to a patient below. In a similar manner, downwash can blow loose rocks and soil from a steep cliff which is not covered by snow. Downwash from a small RPA will not be strong enough to introduce these kinds of hazards, nor will it make the snow swirl up (Table 1). In this way it will be easier to perform a simultaneous search for victims from the ground and from the air.

**Table 1:** Technical specifications rotor-wing RPA versus manned helicopter (EC135 P2+)

Feature	Rotor-wing RPA (prototype config)	EC135 P2+ (HEMS config)
Size	0.84 m × 0.30 m	Length 12.19 m, height 3.51 m
Weight (empty)	1.47 kg	1455 kg
Rotor diameter	0.84 m	10.20 m
Downwash	Negligible	Strong
Noise	Humming	>85 dbA
Max payload	1.53 kg	965 kg
Max air speed	~35 km/h	254 km/h
Power/fuel	Electrical (DC), rechargeable lithium-ion battery	Jet A-1
Max take-off weight	3 kg	2835 kg
Range	Line of sight	Depending on payload and weather conditions, approximately 615 km
Rotary system	6 rotors, 6 DC motors	1 rotor, 2 x Pratt & Whitney PW 206 B2 (463 kW)
Flight endurance	~15 min	166 min (max fuel, ideal weather conditions, 3 crew, standard HEMS configuration)
Operation style	Unmanned, remote controlled	Manned, 3 crew (pilot, HEMS crew member, physician)
Take-off/landing	From landing gear	From landing gear (skid)
Landing site requirement	1 m × 1 m	25 m × 25 m

### Flight safety

Aircraft, both manned and unmanned, will always be at risk of collisions with air obstacles like high voltage cables, high-rise buildings, other aircraft and telephone masts, especially when operating at low altitude and in confined areas. In the event of a major incident, manned helicopter activity might be significant due to the operation in the same area at the same time of Helicopter Emergency Medical Service-, press-, police-, military- and firefighting helicopters [7]. If an unmanned aerial vehicle comes into conflict with a manned aircraft there is a considerable risk that both will crash. Manned helicopter crashes will often be fatal for the crew and are a great danger to people on the ground. Regulations to avoid these kinds of accidents, and to enable manned and unmanned aircrafts to safely share the airspace, are few and differ from country to country [33].

Unmanned aerial vehicles have the potential to be remotely operated beyond line-of-sight. Navigation based solely on visual input from video cameras on board the RPA makes it hard for the RPA pilot to spot other aircraft in the same area due to the limited coverage of images [34]. On the other hand, it is almost impossible to spot a fast-moving small RPA, at no more than 80 cm in diameter, from a manned aircraft. There is no requirement for strobe lights, transponders or traffic collision avoidance systems. The UAV pilot is not mandated to stay in contact with air traffic control in uncontrolled airspace. In most cases the RPA pilot will not be able to communicate with other aircraft or air traffic control because of lack of equipment or insufficient training. Regulation and training of RPA pilots is needed in order not to interfere with manned aircraft in high density flying operations such as major incidents.

Low level flight with an RPA in close proximity to humans can be dangerous. The flying weight of an RPA is only a few kgs, but if it falls down or the fast-rotating rotorblades of the RPA come into contact with human skin, injuries could occur. Flight at night without night vision goggles is feasible but not safe.

### Human factors

The safe operation of an RPA is a difficult task that can be decomposed into individually complex and interacting task steps of a flight operative, technical, communicative and multidisciplinary character [33]. In calm weather, open terrain and at high altitude above the ground, it is feasible to fully operate a UAS using a single pilot, as we did in parts of our simulated scenarios. This is possible with assistance from advanced autonomous features, such as auto-hover, GPS way-point flight, fixed altitude above the ground and automatic take-off and landing. Confined area helicopter operations, windy conditions, limited visibility, dynamic environment and low level flight increase the workload and demands on the pilot. In such cases one or even two additional operators will be needed to help out with interpreting images on the ground station monitor and directing the RPA into a position to appropriately frame the video [34].

Efficient allocation of scarce resources will be a trade-off between responders needed in the field opposed to human resources needed to operate the RPA.

### Ethical issues

Real-time aerial video capture from a major incident scene raises a large number of ethical and legal issues. In essence, this kind of collecting of video and images of patients and rescue service operators can be categorised as telemedicine and must adhere to the fundamental medicolegal principles of telemedical care [35]. Firstly, patient care and incident management need not be disturbed. We face substantial challenges regarding the protection of privacy and confidentiality for both patients and bystanders. Obtaining informed consent from patients or bystanders will at best be difficult but in most cases, not possible at all. Children, unconscious- and heavily injured patients lack the capacity to consent. Video recordings from a remotely piloted, unmanned, aerial vehicle have the potential to be streamed to a large number of ground stations and people in real time, and recordings of mass casualty incidents may contain video clips of many patients at the same time. Professional groups involved in the rescue operation could benefit from live video feed in various ways but need to extract different information from the same recording. It is not at all clear who will have the ownership of these recordings and who should have access to them. The content will be of great value for secondary purposes such as research, teaching and training. Videos of disaster areas and rescue operations will most likely be of public and commercial interest. The transmission of real-time video must be encrypted to avoid leakage. Video recordings have to be anonymised, treated and stored as part of a patient’s medical record. The same standards of confidentiality, and the same requirements for consent to disclosure, apply.

### Limitations

We used a custom-made, rotor-wing, remotely piloted aircraft system, which is not commercially available. Performance was restricted by the inherent technological limitations of the RPA available. We did not test the use of an unmanned fixed wing aircraft or multiple RPAs at the same time.

The resolution of the video that was downlinked to the ground control station was not high definition. Signal noise reduced image quality on the screen and it was not possible to demonstrate bidirectional telecommunication between the RPA pilot and rescue services in the field.

Major incidents and emergency medical missions in disaster areas are exceedingly variable in nature, and both environmental and weather conditions differ considerably.

Our RPA was used in a small number of simulated scenarios with simulated patients only, and the weather was excellent for RPA operation in all scenarios. The terrain and the geographical span of the simulated incidents allowed for line-of-sight operation.

We have not explored unintended and undesirable consequences of introducing new advanced technology in an already technology-intensive work environment.

### Future research

Firstly, we need to determine how real-time aerial video stream from a major incident will impact on decision-making and situation assessment. In order to avoid introducing hazards in an already error-prone environment, and to avoid a negative impact on how teams perform, a human factors and ergonomics approach will probably be appropriate when considering the implementation of this powerful technology. Hierarchical task analysis can provide an understanding of human-human and human-machine interactions [36]. We also need to explore the kind of situations in which it can be optimal to deploy an RPA, and how the information it provides can be used in the best possible way to support situation assessment and decision-making. We need to explore whether RPAs can be used as a tool to gather data in medical research and in simulation-based exercises.

## Conclusions

It is feasible to use a rotor-wing RPA as a tool carrier for light goods and as a flying platform for audiovisual equipment and sensors in pre-hospital environments, despite its limited payload capacity and flight endurance. It is also feasible to shoot high resolution, aerial video from a remotely piloted unmanned aircraft and to distribute this video wirelessly in real time. Avalanche beacon search and remote aerial visual search from an RPA are feasible. Incident command, rescue workers and emergency medical coordination centres can use this supplemental visual information to support situation assessment, decision-making and information exchange in major incidents. Ethical issues are abundant.

## Additional files

Additional file 1:
**Aerial video of search and rescue operation shot from a remotely piloted aircraft (Quicktime).** Air view from different hights of narrow canyon with injured skier located at the bottom of a steep rock face with overhanging snowdrifts. Neither noise nor downwash distracts the rescue workers. Note the big hole in the snow at the bottom of the canyon with running water beneath, not visible from the edge. (RPA-video: André Kjellstrup). Additional file 2:
**A video shot from the ground (Quicktime) showing a simulated avalanche beacon search from low altitude using a manned, Norwegian air ambulance helicopter (EC 135).** An avalanche receiver (black and yellow) is carried like an underslung load at low altitude above a simulated avalanche in Sirdalen, Norway. Communication between rescue workers on the ground is almost impossible because of the loud noise and strong downwash from the helicopter. (Video: Håkon B. Abrahamsen).

## Abbreviations

RPA: Remote Piloted Aircraft
NCAA: Norwegian Civil Aviation Authority
PLA: Poly Lactic Acid
GCS: Ground Control Station
UAV: Unmanned Aerial Vehicle
UAS: Unmanned Aerial System
SD: Secure Digital
